# Osmotic Dehydration Model for Sweet Potato Varieties in Sugar Beet Molasses Using the Peleg Model and Fitting Absorption Data Using the Guggenheim–Anderson–de Boer Model

**DOI:** 10.3390/foods13111658

**Published:** 2024-05-25

**Authors:** Lato Pezo, Biljana Lončar, Vladimir Filipović, Olja Šovljanski, Vanja Travičić, Jelena Filipović, Milada Pezo, Aca Jovanović, Milica Aćimović

**Affiliations:** 1 Institute of General and Physical Chemistry, University of Belgrade, Studentski Trg 12–16, 11000 Belgrade, Serbia; akiyov@gmail.com; 2 Faculty of Technology Novi Sad, University of Novi Sad, 21000 Novi Sad, Serbia; cbiljana@uns.ac.rs (B.L.); vladaf@uns.ac.rs (V.F.); oljasovljanski@uns.ac.rs (O.Š.); vanjaseregelj@uns.ac.rs (V.T.); 3 Institute of Food Technology, University of Novi Sad, Bulevar Cara Lazara 1, 21000 Novi Sad, Serbia; jelena.filipovic@fins.uns.ac.rs; 4 Department of Thermal Engineering and Energy, “VINČA” Institute of Nuclear Sciences—National Institute of the Republic of Serbia, University of Belgrade, Mike Petrovića Alasa 12–14, 11351 Belgrade, Serbia; milada@vinca.rs; 5 Department of Vegetable and Alternative Crops, Institute of Field and Vegetable Crops, 21000 Novi Sad, Serbia; milica.acimovic@ifvcns.ns.ac.rs

**Keywords:** Peleg model, osmotic dehydration, sweet potato samples, sugar beet molasses, mass transfer kinetics, sorption isotherms, Guggenheim, Anderson, and de Boer (GAB) model, effective moisture diffusivity, activation energy

## Abstract

This study investigates the applicability of the Peleg model to the osmotic dehydration of various sweet potato variety samples in sugar beet molasses, addressing a notable gap in the existing literature. The osmotic dehydration was performed using an 80% sugar beet molasses solution at temperatures of 20 °C, 35 °C, and 50 °C for periods of 1, 3, and 5 h. The sample-to-solution ratio was 1:5. The objectives encompassed evaluating the Peleg equation’s suitability for modeling mass transfer during osmotic dehydration and determining equilibrium water and solid contents at various temperatures. With its modified equation, the Peleg model accurately described water loss and solid gain dynamics during osmotic treatment, as evidenced by a high coefficient of determination value (r^2^) ranging from 0.990 to 1.000. Analysis of Peleg constants revealed temperature and concentration dependencies, aligning with previous observations. The Guggenheim, Anderson, and de Boer (GAB) model was employed to characterize sorption isotherms, yielding coefficients comparable to prior studies. Effective moisture diffusivity and activation energy calculations further elucidated the drying kinetics, with effective moisture diffusivity values ranging from 1.85 × 10^−8^ to 4.83 × 10^−8^ m^2^/s and activation energy between 7.096 and 16.652 kJ/mol. These findings contribute to understanding the complex kinetics of osmotic dehydration and provide insights into the modeling and optimization of dehydration processes for sweet potato samples, with implications for food processing and preservation methodologies.

## 1. Introduction

The sweet potato is a globally significant food crop, especially with Asia, particularly China, leading in production, followed by Africa, valued for its storage roots rich in dietary energy and essential nutrients, including beta-carotene and vitamin C [1,2]. The color of sweet potato skins and flesh can vary from white, cream, yellow, orange, pink, and red to purple [3]. Due to their seasonal nature and the need to utilize them within a limited timeframe post-harvest, sweet potatoes possess a short storage period characterized by high moisture content, so drying preservation methods are needed [4]. For sweet potato preservation, different drying methods have been utilized, including air drying [5], drum drying [6], microwave drying [7], microwave vacuum drying [8], freeze-drying [9], and infrared freeze drying [10]. Various pretreatments can enhance drying efficiency, including microwave and blanching pretreatments [11], hot water blanching and saline immersion [12], ultrasonic pretreatments [10], and osmotic dehydration [13]. Using osmotic pretreatment may alter the structure, appearance, and taste of dehydrated sweet potatoes, affecting the effectiveness of water permeation during the drying phases [14]. Choosing the appropriate hypertonic solution is essential for enhancing the quality of the dehydrated product [15,16]. Sugar beet molasses has been recognized as a beneficial osmotic solution for osmotic dehydration of both plant and animal-origin food products [17,18,19].

The mass transport mechanisms from osmotic solution to sample and vice versa involved during osmotic dehydration are complex; therefore, theoretical, semi-theoretical, and semi-empirical models were utilized to characterize them [20,21].

In traditional sorption isotherm experiments, samples are placed in a chamber with a known vapor pressure and constant temperature until equilibrium is reached [22]. Moisture content is then measured and plotted against water activity. In the osmotic dehydration study, samples are immersed in a liquid osmotic medium at different temperatures and durations, with water activity measured separately at room temperature (23 °C) [23].

Numerous empirical and semi-empirical formulations have been suggested to establish the relationship between the equilibrium moisture content and the water activity of food items. Among these, the GAB (Guggenheim–Anderson–de Boer) equation has demonstrated successful application across various food types [24] and is endorsed by [25]. The GAB model comprises three constants with physical significance, two of which vary with temperature.

Application of the GAB model to water adsorption isotherms for dried raisins posed some challenges, necessitating linear regression analysis [26]. Dried fruits, abundant in sugars (monosaccharides), exhibit distinctive sorption isotherm shapes characterized by low moisture content at low water activities and sudden moisture increases at high water activities. In a study examining the storage stability of dried fruits, adsorption isotherms for dried raisins, figs, prunes, and apricots were determined at temperatures of 15, 30, 45, and 60 °C [27].

The GAB model is based on desorption isotherms and can be effectively used to predict the water activity (*a_w_*) of a food product during osmotic dehydration using moisture content data, thereby reducing the need for extensive experimental work. Additionally, this model can be integrated into existing models for describing water transport during dehydration, enabling direct prediction of water activity in the product [23].

Linear regression of the GAB model yielded less accurate results compared to non-linear regression due to the challenges in transforming the GAB model into a linear equation with respect to the three constants, resulting in significant mean relative errors between experimental and predicted equilibrium moisture content values [28]. Nevertheless, the GAB model proved effective in accurately describing sorption isotherms for ground coffee within the temperature range of 20 to 80 °C [29]. However, its predictive ability concerning the temperature’s influence on adsorption isotherms for dried raisins was less satisfactory, presumably due to the dissolution of fruit sugars at high water activities [26].

Peleg [30] introduced a two-parameter sorption equation and assessed its accuracy in predicting water adsorption in milk powder and whole rice, as well as the soaking of whole rice. Palou, Lopez-Malo, Argaiz, and Welti [31] investigated simultaneous water desorption and sucrose absorption during the osmotic dehydration (OD) of papaya using the Peleg model. Notably, there is a gap in the existing literature regarding the utilization of the Peleg model for OD in sugar beet molasses solutions, i.e., only a few works were found in the open literature [32].

This study aimed to address this gap by achieving the following two main objectives: (i) evaluating the suitability of the Peleg equation in modeling mass transfer during OD of sweet potato samples, including orange, white, pink, and purple varieties, in sugar beet molasses solution; and (ii) determining the equilibrium water and solid contents for OD at various sweet potato samples and temperatures. Using Peleg’s equation as a starting point, a robust and simplified mathematical model could be developed for predicting water loss (WL) during the osmotic dehydration of sweet potatoes in sugar beet molasses. The sorption isotherms were characterized using the GAB model, resulting in coefficients that align with previous research. Calculations of effective moisture diffusivity and activation energy provided additional insights into the drying kinetics. The effective moisture diffusivity values were found to range from 1.85 × 10^−8^ to 4.83 × 10^−8^ m^2^/s, while the activation energy values ranged between 7.096 and 16.652 kJ/mol.

## 2. Materials and Methods

### 2.1. Osmotic Treatment

Various sweet potatoes were sourced from a family farm specializing in their cultivation in Kać, Novi Sad, and the Republic of Serbia (coordinates: 45.2993319° N, 19.9462154° E). This study utilized four distinct sweet potato varieties, distinguished primarily by flesh color: white, pink, orange, and purple. The edible portions were isolated (peeled) and subjected to osmotic dehydration using sugar beet molasses. The sweet potatoes were sliced into pieces measuring 5 cm in thickness and 3.6 cm in diameter using a Nemco slicer (model: 55200AN, Hicksville, OH, USA), with an accuracy of ±0.27 mm [33]. Osmotic pre-treatment was conducted in an 80% sugar beet molasses solution at temperatures of 20 °C, 35 °C, and 50 °C for durations of 1, 3, and 5 h. The ratio of sample/solution was 1:5. The samples were manually stirred every 15 min.

Following the designated osmotic pre-treatment periods, the samples were rinsed with distilled water, gently dried with tissue paper, and then measured.

The calculations of osmotic parameters during the osmotic treatment of sweet potatoes were conducted according to the following equations (Mišljenović et al.) [34]:

(1) $$SG=\frac{u-u_{0}}{w_{0}},$$

Moreover, *WL* could be calculated according to Equation (2), Assis et al. 2017 [35], as follows:

(2) $$WL=\frac{w_{w0}-w_{w}}{w_{0}}$$

where *w*_0_ is the initial weight of the sweet potato sample (g), *u*_0_ is the weight of dry matter in the fresh sweet potato sample (g), *u* is the weight of dry matter in the sweet potato sample after osmotic dehydration (g), *w_w_*_0_ is the initial moisture content in the sweet potato sample (g), and *w_w_* is the moisture content in the sweet potato sample at time *t* (g).

### 2.2. Peleg Model

Peleg [30] introduced an equation represented as follows (3):

(3) $$X_{w}=X_{w0}\pm \frac{t}{k_{1}+k_{2}\cdot t}$$

In this equation, *X_w_* signifies the moisture content at time *t*, expressed on a dry basis; *X_w0_* represents the initial moisture content, also on a dry basis; *k*_1_ (h·g/g) denotes the Peleg rate constant, while *k*_2_ (g/g) is the Peleg capacity constant. In this study, the Peleg equation was modified to incorporate *WL* or *SG* instead of moisture content. Subsequently, the variables were denoted as *Y*, where *Y* could be either *WL* or *SG*.

(4) $$Y=\frac{t}{k_{1}^{Y}+k_{2}^{Y}\cdot t}$$

where $k_{1}^{Y}$ and $k_{2}^{Y}$ are the Peleg constants for *WL* or *SG*.

The Peleg rate constant $k_{1}^{Y}$ pertains to the initial dehydration rate at *t* = 0.

(5) $${\left(\frac{dY}{dt}\right)}_{t\to 0}=\frac{1}{k_{1}^{Y}}$$

The Peleg capacity constant $k_{2}^{Y}$ is associated with the minimum achievable moisture content. As *t* approaches infinity, Equation (4) establishes the relationship between equilibrium values *WL*_∞_ or *SG*_∞_ and $k_{2}^{Y}$. At equilibrium, Peleg’s equation for *WL* and *SG* is as follows:

(6) $$Y_{\infty }=\frac{1}{k_{2}^{Y}}$$

The Peleg model offers a significant advantage by accurately predicting the water sorption kinetics of foods, including the equilibrium moisture content, using concise experimental data and thus saving time.

### 2.3. Statistical Analyses

The data were subjected to statistical analysis using Statistica 10.0 software (StatSoft Inc., Tulsa, OK, USA). The results obtained are presented as the mean value, accompanied by the standard deviation (SD). To examine the variations in the observed parameters, an analysis of variance (ANOVA) was conducted, followed by Tukey’s HSD post hoc test for comparing sample means. Additionally, all observed samples were assessed for variance equality using Levene’s test and checked for normal distribution using the Shapiro-Wilk’s test.

### 2.4. Mathematical Modeling of Drying Curves

The moisture ratio (MR) of sweet potato samples during the drying process was calculated based on the equation proposed by Doymaz [36] and Šobot et al. [37], as seen below.

(7) $$MR=\frac{M-M_{e}}{M_{0}-M_{e}}$$

where *M*, *M*_0_, and *M_e_* are the moisture content at any time of drying, the initial moisture content, and the equilibrium moisture content, respectively.

The obtained drying data were used for the determination of diffusivity coefficients using Fick’s second diffusion model, Doymaz [36], as follows:

(8) $$\frac{\partial M}{\partial t}=D_{eff}\cdot \nabla^{2}M$$

It was assumed that the change in moisture volume occurred in a unidimensional manner [38]. Under this assumption, the analytical solution for Equation (2) could be derived for the drying process of sweet potato samples. Assuming constant temperature and diffusivity coefficients and negligible external resistance, the moisture ratio could be expressed as per 2nd Fick’s law [39] as follows:

(9) $$MR=\frac{8}{\pi^{2}}\cdot \exp\left(-\frac{\pi^{2}\cdot D_{eff}\cdot t}{4\cdot L^{2}}\right)$$

The effective diffusivity can be calculated according to temperature by an Arrhenius-like expression [36], as follows:

(10) $$\ln\left(D_{eff}\right)=\ln\left(D_{0}\right)-\frac{E_{a}}{R\cdot \left(T+273.15\right)}$$

where *D*_0_ is the constant in the Arrhenius equation (m^2^/s), *E_a_* is the activation energy (kJ/mol), *T* is the temperature of the air (°C), and *R* is the universal gas constant (8.314 kJ/mol∙K).

The natural logarithm of the calculated effective diffusion coefficient (*D_eff_*) was plotted against the reciprocal of the absolute temperature. Within the range of temperatures studied, this graph formed a straight line, indicating the presence of an Arrhenius relationship between the variables. The activation energy was determined by analyzing the slope of this linear relationship, and this analysis was conducted using Microsoft Excel 2016.

### 2.5. Moisture Isotherms Model

The sorption isotherms describe the equilibrium relationship between the moisture content of a material and the relative humidity of the surrounding environment at a constant temperature [40]. Sorption isotherms are crucial for understanding the hygroscopic properties of materials and their interaction with moisture. However, osmotic dehydration could not be treated as the final step in the drying process (the obtained product is not microbiologically safe due to its high *a_w_* value) [41].

Sorption isotherms were constructed by experimentally determining the equilibrium moisture content of a material at various levels of relative humidity [42]. The material was exposed to controlled humidity conditions until equilibrium was reached, then measured for moisture content. The resulting data were plotted to form the isotherm curve, which was analyzed using models like the Guggenheim, Anderson, and de Boer (GAB) model [43].

The importance of obtaining accurate sorption isotherm data lies in its application to drying kinetics, storage stability, and product quality control. Understanding the moisture sorption behavior of a material allows for the optimization of drying processes, the prediction of shelf life, and the prevention of moisture-related degradation. The effective moisture diffusivity and activation energy derived from these isotherms further elucidate the drying kinetics, helping to design more efficient drying protocols and ensuring the preservation of product integrity [44]. The Guggenheim, Anderson, and de Boer (GAB) equation was employed to represent the dry basis moisture content (*x*) as a function of water activity (*a_w_*) [23], as follows:

(11) $$x=\frac{X_{m}\cdot C\cdot K\cdot a_{w}}{\left(1-K\cdot a_{w}\right)\cdot \left(1-K\cdot a_{w}+C\cdot K\cdot a_{w}\right)}$$

where *x* is the moisture content (dry-based); *a_w_* is the water activity; *X_m_*, *K*, and *C* are three free sorption parameters characterizing the sorption properties of the material; *C* and *k* are constants; and *X_m_* is described in the literature as the monolayer moisture content on a dry basis (kg/kg).

### 2.6. Error Analysis

In terms of error analysis, the accuracy of the developed models was evaluated through several key metrics, i.e., coefficient of determination (*r*^2^), reduced chi-square (*χ*^2^), mean bias error (*MBE*), root mean square error (*RMSE*), mean percentage error (*MPE*), the sum of squared errors (*SSE*), and average absolute relative deviation (*AARD*). These widely used parameters were employed to assess the validity of the models [45], as follows:

(12) $$\chi^{2}=\frac{\sum_{i=1}^{N}{\left(x_{\exp,i}-x_{pre,i}\right)}^{2}}{N-n},$$

(13) $$RMSE={\left[\frac{1}{N}\cdot \sum_{i=1}^{N}{\left(x_{pre,i}-x_{\exp,i}\right)}^{2}\right]}^{1/2},$$

(14) $$MBE=\frac{1}{N}\cdot \sum_{i=1}^{N}\left(x_{pre,i}-x_{\exp,i}\right),$$

(15) $$MPE=\frac{100}{N}\cdot \sum_{i=1}^{N}\left(\frac{\left|x_{pre,i}-x_{\exp,i}\right|}{x_{\exp,i}}\right)$$

(16) $$SSE=\sum_{i=1}^{N}{\left(x_{pre,i}-x_{\exp,i}\right)}^{2}$$

(17) $$AARD=\frac{1}{N}\cdot \sum_{i=1}^{N}\left|\frac{x_{exr,i}-x_{pre,i}}{x_{exr,i}}\right|$$

where *x_exp,i_* stands for the experimental values; *x_pre,i_* are the predicted values calculated by the model; and *N* and *n* are the number of observations and constants, respectively.

## 3. Results and Discussion

### 3.1. Experimental Results

**Figure 1:**
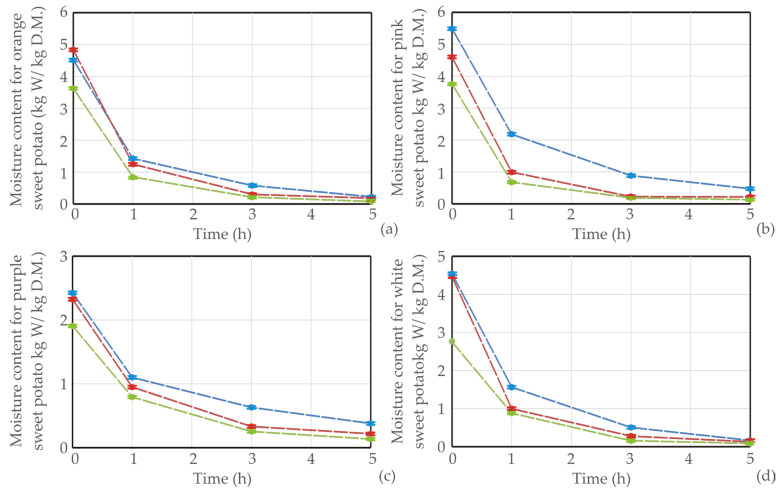
Graphical presentation of moisture content (kg water/kg dry matter) during OD of (**a**) orange, (**b**) pink, (**c**) purple, and (**d**) white sweet potato samples in sugar beet molasses at different temperatures (blue line for 20 °C, red for 35 °C, and green line for 50 °C), *n* = 3 measurements.

**Figure 2:**
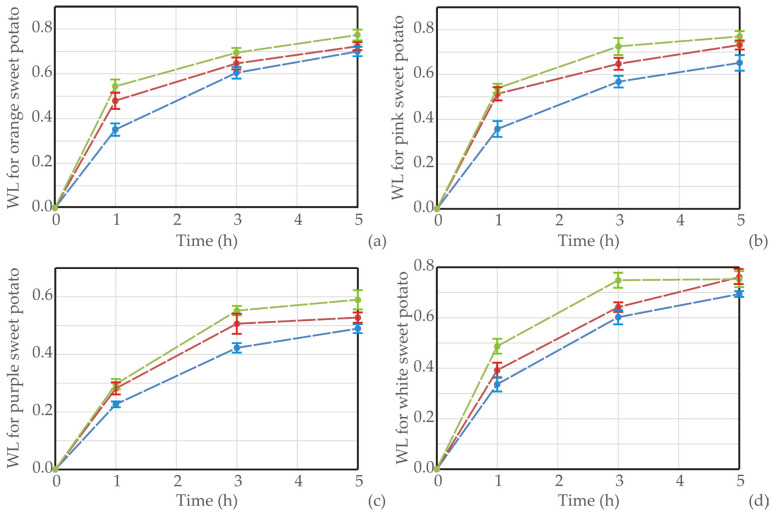
Graphical presentation of *WL* during OD of (**a**) orange, (**b**) pink, (**c**) purple, and (**d**) white sweet potato samples in sugar beet molasses at different temperatures (blue line for 20 °C, red for 35 °C, and green line for 50 °C), *n* = 3 measurements.

**Figure 3:**
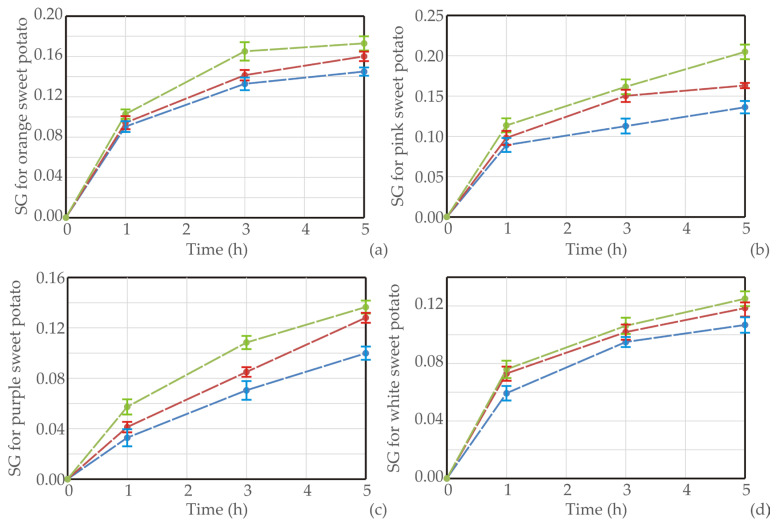
Graphical presentation of *SG* during OD of (**a**) orange, (**b**) pink, (**c**) purple, and (**d**) white sweet potato samples in sugar beet molasses at different temperatures (blue line for 20 °C, red for 35 °C, and green line for 50 °C), *n* = 3 measurements.

Experimental results of the drying process for four sweet potato varieties were presented in Figure 1, Figure 2 and Figure 3, where the moisture content, *WL,* and *SG* of samples were recorded according to different temperatures.

The experimental data for water loss (*WL*) and solid gain (*SG*) during the osmotic dehydration (OD) of various sweet potato types at different operating temperatures were obtained. Throughout the process, a nonlinear increase in both *WL* and *SG* was noted across all sweet potato types and temperatures. The rise in temperature had a positive impact on dehydration efficiency, leading to increased *WL* and decreased *SG*. All sweet potato types revealed an initial rapid water removal and solid uptake throughout the immersion period, followed by a slower phase in the later stages. This rapid initial water loss and solid gain can be attributed to the substantial osmotic driving force between the fresh sweet potato cubes in the hypertonic medium (sugar beet molasses).

Similar results were obtained in the study of Mandala et al. [46]. The SG and WL values of osmotically treated apples were influenced by immersion time, sugar concentration, and sugar type. Moreover, during drying in an osmotic solution, a significant moisture decline initially occurs, but a slighter decrease in the moisture ratio is noted at longer drying times. This phenomenon indicates that the system is approaching the end of the osmotic process, reaching a pseudo-equilibrium state [47]. Notably, the highest *WL* (0.773 g/g of initial sample weight) was observed in the orange sweet potato sample dehydrated in molasses at 50 ºC for 5 h [48].

**Figure 4:**
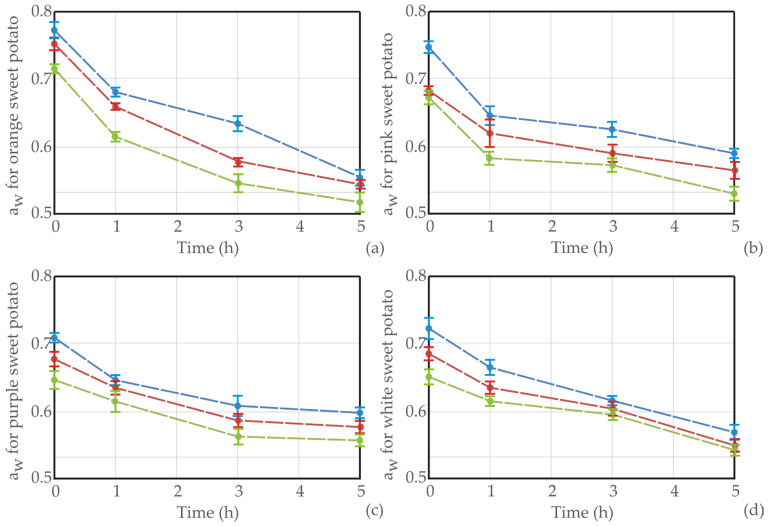
Graphical presentation of *a_w_* during OD of (**a**) orange, (**b**) pink, (**c**) purple, and (**d**) white sweet potato samples in sugar beet molasses at different temperatures (blue line for 20 °C, red for 35 °C, and green line for 50 °C), *n* = 3 measurements.

Figure 4 presents *a_w_* values on osmotic dehydration in sugar beet molasses for various sweet potato types at different time intervals and temperatures. Across all sweet potato types, as the dehydration time increases from 0 to 5 h, the water activity (*a_w_*) generally decreases. Additionally, variations in *a_w_* are observed among different sweet potato types at the same dehydration time and temperature. At a temperature of 20 °C, orange sweet potatoes exhibit *a_w_* values ranging from 0.553 to 0.681, while pink, purple, and white sweet potatoes show *a_w_* ranges ranging from 0.590 to 0.747, 0.580 to 0.649, and 0.533 to 0.673, respectively. At higher temperatures, such as 50 °C, the *a_w_* values generally decrease compared to those at lower temperatures, indicating more effective dehydration. This conclusion is supported by the findings of Yadav and Singh [49], who reported that the water loss and water activity of the final product depend not only on the a_w_ of the osmotic solution but also on the amount of solids in the sample. Furthermore, the quality of the final product is influenced by the treatment process, solid gain, chemical composition of the solution, and the shape of the sample.

### 3.2. Peleg’s Model

Experimental data from *WL* and *SG* were used to evaluate the adequacy of the Peleg equation. The coefficient of determination values, *r*^2^, varied from 0.990–1.000 for both *WL* and *SG*. Such high values of *r*^2^ indicate a good fit to the experimental data and suggest that the Peleg equation adequately describes the mass transfer kinetics terms during the osmotic dehydration of sweet potatoes in sugar beet molasses.

Table 1 displays the values of *k*_1_ and *k*_2_ for various sweet potato types and temperatures. The reciprocal of *k*_1_ characterizes the initial mass transfer rate; a lower *k*_1_ indicates a higher mass transfer rate. The data in Table 1 reveals that, at a constant temperature, *k*_1_ for *WL* and *SG* decreases with an increase in temperature. This trend aligns with the influence of concentration on *WL* and *SG*, as demonstrated in Figure 1. Both *k*_1_ values for *WL* and *SG* decrease as the temperature rises, a pattern consistent with the observations made by Corozo and Bracho [50] and Ganjloo et al. [51]. Additionally, Table 1 presents the Peleg capacity constants for *WL* and *SG*. These constants are associated with *WL* and *SG*; lower *k*_2_ values correspond to higher *WL* or *SG*. Specifically, *k*_2_ for *WL* decreases with increasing concentration, *k*_2_ for *SG* increases with concentration, and both *k*_2_ values for *WL* and *SG* decrease with higher temperatures.

The initial dehydration rate (*k*_1_) for orange sweet potatoes decreases as the temperature increases from 20 °C to 50 °C. Water loss (*WL*) decreases with increasing temperature [52].

Purple sweet potatoes exhibit higher initial dehydration rates (*k*_1_) compared to orange and pink varieties. Water loss (*WL*) tends to decrease with increasing temperatures.

Initial dehydration rates for white sweet potatoes (*k*_1_) show a decreasing trend with increasing temperature. Water loss (*WL*) decreases with increasing temperature [52].

To estimate the equilibrium values of *WL* and *SG* (as shown in Table 2), Equation (4) was utilized. Equilibrium is achieved when the water activity of the sweet potato samples and the solution are equal. Both *WL* and *SG* contribute to the reduction in water activity, indicating that the relationship between these two phenomena plays a crucial role in reaching the equilibrium point, as also noted by Corozo and Bracho [50].

Generally low values across different goodness of fit indices for orange sweet potatoes indicate good model performance. RMSE values range from 4.26 × 10^−4^ to 1.13 × 10^−2^, indicating relatively small errors. The coefficient of determination (r^2^) was consistently close to 1.000, indicating a strong fit of the model to the data.

Similar to orange sweet potatoes, generally low values were observed across goodness-of-fit indices for pink sweet potatoes. RMSE values range from 2.14 × 10^−3^ to 1.41 × 10^−2^, indicating relatively small errors, while the coefficient of determination (r^2^) is consistently close to 1, indicating a strong fit of the model to the data.

Slightly higher model errors were observed for purple sweet potatoes compared to orange and pink sweet potatoes. RMSE values range from 4.91 × 10^−3^ to 1.80 × 10^−2^, indicating slightly larger errors. The coefficient of determination (r^2^) is consistently high, indicating a good fit of the model to the data.

Generally low errors, similar to orange and pink sweet potatoes, were observed for white sweet potatoes. RMSE values range from 4.89 × 10^−3^ to 2.31 × 10^−2^, indicating relatively small errors. The coefficient of determination (r^2^) is consistently close to 1, indicating a strong fit of the model to the data.

### 3.3. GAB Model

In line with existing literature [53,54], the Guggenheim, Anderson, and de Boer (GAB) model frequently serves as a descriptor for sorption isotherms.

The GAB model encompasses not only the apparent parameters (*X_m_*, *K*, and *C*) but also factors in temperature dependence through these parameters. The regression coefficients in the GAB model are presented in Table 3. Within this investigation, the equation parameters were estimated using the generalized reduced gradient algorithm (Microsoft Excel Solver) for nonlinear problems. This method optimizes the three parameters by minimizing the sum of the residual errors. The sum of squares was calculated to assess the fit of the model at each temperature. Due to the challenge of obtaining a unique solution, the regression procedure was repeated with various initial values and different upper and lower limits until stable and reproducible values were obtained.

In comparison to previous studies [55], the results for sweet potatoes are acceptable, demonstrating similar values for GAB model coefficients (*X_m_*, *K*, and *C*).

Comparing the obtained results with those of Liendo-Cardinas et al. [56], there were no observations of the inversion of isotherms within the water activity range of 0.80–0.92. The authors attribute this to sugar dissolution in water. However, in studies such as Kechaou and Maalej [57] on dates, which contain a higher sugar content than sweet potatoes, this inversion is not evident at higher water contents. Similar conclusions are drawn in the study conducted by Touil et al. [58].

Fluctuations between different varieties of sweet potato were observed in fats, total sugars, cellulose, ash, and total carbohydrate contents [30]. For orange sweet potatoes, there are generally consistent values of *Ck*, *k*, and *Xm* across different temperatures. Pink sweet potatoes show similar trends to orange sweet potatoes, with slight variations in the values of *Ck*, *k*, and *Xm*. The osmotic dehydration of purple sweet potatoes caused significant variations, particularly in moisture content and total carbohydrates. Purple sweet potatoes exhibit consistent values of *Ck*, *k*, and *Xm* across different temperatures, indicating stable moisture adsorption characteristics. White sweet potatoes display slight variations in the values of *Ck*, *k*, and *Xm* across different temperatures, with relatively higher values compared to other sweet potato types.

Table 4 displays the verification results of GAB models for different types of sweet potatoes at varying temperatures. For orange sweet potatoes, there is a consistent trend of decreasing errors and increasing coefficients of determination (r^2^) with increasing temperature. Similarly, pink sweet potatoes exhibit decreasing errors and increasing r^2^ values as the temperature rises. Purple sweet potatoes display relatively low errors across temperatures, with r^2^ values consistently close to 1, indicating a strong fit of the model. White sweet potatoes also demonstrate decreasing errors with increasing temperature, with r^2^ values consistently close to 1, suggesting a good fit of the model. Overall, the GAB models perform well across different types of sweet potatoes and temperatures, with relatively low errors and high coefficients of determination.

### 3.4. Determination of the Effective Moisture Diffusivity

**Figure 5:**
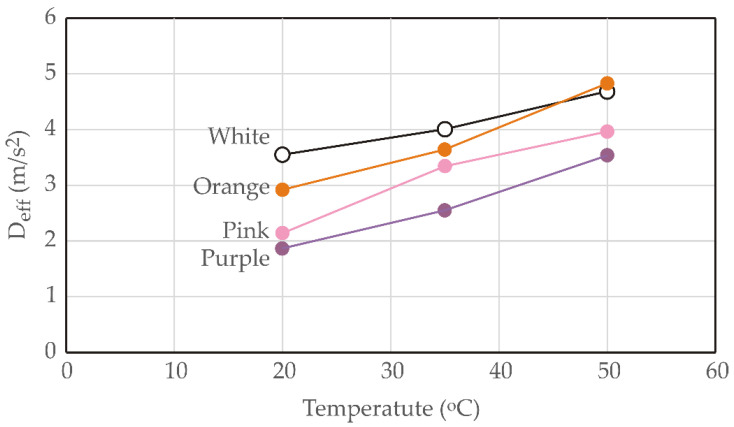
Effective diffusivity (*D_eff_*) for orange, pink, purple, and white sweet potato samples at various temperatures.

Figure 5 presents the estimated values of effective diffusivity (*D_eff_*) for orange, pink, purple, and white sweet potato samples at various temperatures. During the drying phase, *D_eff_* values ranged from 1.85 × 10^−8^ to 4.83 × 10^−8^ m^2^/s.

Notably, as the temperature increased, the *D_eff_* values exhibited a significant rise. The *D_eff_* values obtained in this study fall within the general range of 10^−12^ to 10^−8^ m^2^/s, which is commonly observed in the drying of food materials [59]. These findings align well with existing literature, where comparable results were reported, such as 7.118 × 10^−10^ to 1.359 × 10^−9^ m^2^/s for the osmotic dehydration of tomato samples in ternary solutions [60].

Figure 5 presents the major results for sweet potato samples, including orange, pink, purple, and white varieties, at different temperatures. As the temperature increases from 20 °C to 50 °C, there is a noticeable trend of increasing values for the effective diffusion coefficient (*D_eff_*) across all sweet potato types. For orange sweet potatoes, *D_eff_* values increase from 2.92 × 10^−10^ m^2^/s at 20 °C to 4.83 × 10^−10^ m^2^/s at 50 °C. Similarly, pink sweet potatoes exhibit a rise in *D_eff_* values from 2.14 × 10^−10^ m^2^/s to 3.96 × 10^−10^ m^2^/s across the same temperature range. Purple sweet potatoes also show an increase in *D_eff_* values from 1.85 × 10^−10^ m^2^/s to 3.54 × 10^−10^ m^2^/s, while white sweet potatoes demonstrate a rise from 3.55 × 10^−10^ m^2^/s to 4.68 × 10^−10^ m^2^/s. The differences in *D_eff_* values could be attributed to the variation in chemical composition of the samples [33].

### 3.5. Activation Energy

The activation energy for four investigated sweet potato samples of *D_eff_* at different temperatures is presented in Table 5. The activation energy values obtained for the sweet potato samples, ranging from 7.096 to 16.652 kJ/mol, are crucial indicators of the energy required to initiate the drying process effectively. The activation energy values obtained for the different sweet potato samples show variations among the samples.

From the straight line’s slope described by the Arrhenius equation, the activation energy was found to be between 7.096 and 16.652 kJ/mol. The obtained values were consistent with literature data, where the activation energy for osmotically treated dried white-flesh cassava (*Manihot esculenta*) ranged from 10.1 to 13.3 kJ/mol [61].

## 4. Conclusions

This study delved into the applicability of Peleg’s model for osmotic dehydration using various sweet potato varieties in sugar beet molasses, filling a significant gap in the existing literature. Through meticulous experimentation and analysis, several key findings emerged. The modified Peleg’s equation effectively modeled mass transfer dynamics during osmotic treatment, exhibiting high coefficient of determination values (r^2^) ranging from 0.990 to 1.000. The Peleg constants demonstrated temperature and concentration dependencies, aligning well with previous observations. The Guggenheim, Anderson, and de Boer (GAB) model was employed to characterize sorption isotherms, yielding coefficients comparable to prior studies. However, challenges were encountered, particularly in the linear regression analysis, due to the dried sweet potato’s unique sorption isotherm shapes.

According to drying kinetics results, moisture diffusivity values ranged from 1.85 × 10^−8^ to 4.83 × 10^−8^ m^2^/s and activation energy between 7.096 and 16.652 kJ/mol for the observed sweet potato samples.

The GAB model demonstrated a good fit with experimental data for all studied temperatures, particularly being suitable for water activities between 0.519 and 0.750 and temperatures between 20 °C and 50 °C. A strong correlation was observed between the *C_k_* constant in the GAB model and temperature. However, it is important to note that the GAB model is empirical and not derived from any physical laws or diffusion theories. Therefore, despite its demonstrated success, its general applicability cannot be assumed and must be independently established for each specific system. The model can be helpful for comparing the behavior of different food products, osmotic solutions, and processing methods in reducing water activity during osmotic dehydration, thereby enhancing the understanding of the osmotic process.

The presented results greatly enhance the grasp of the intricate dynamics involved in osmotic dehydration, providing valuable insights for improving the dehydration processes for sweet potato samples. They highlight the crucial role of precise modeling and analysis in enhancing food processing and preservation techniques.

## Figures and Tables

**Table 1:** Peleg rate (*k*_1_) and capacity (*k*_2_) constants and goodness of fit of the Peleg model under different conditions of molasses concentration and temperature (all $k_{1}^{WL}$, $k_{2}^{WL}$, $k_{1}^{SG}$, $k_{2}^{SG}$, initial dehydration rate, and equilibrium *WL* and *SG*. All presented values are significant at the *p* < 0.05 level).

	Sweet Potato		*k*_1_ (h·g/g)	C.I. (−95%)	C-I-(+95%)	*k*_2_ (g/g)	C.I. (−95%)	C.I. (+95%)	Initial Dehydration Rate	Equilibrium WL/SG
*WL*	Orange	20	1.767 ± 0.045	1.655	1.879	1.073 ± 0.004	1.062	1.084	0.566	0.932
		35	0.879 ± 0.007	0.862	0.896	1.226 ± 0.038	1.132	1.319	1.138	0.816
		50	0.676 ± 0.012	0.648	0.705	1.180 ± 0.018	1.135	1.225	1.479	0.847
	Pink	20	1.595 ± 0.046	1.480	1.711	1.220 ± 0.026	1.154	1.285	0.627	0.820
		35	0.718 ± 0.020	0.667	0.768	1.254 ± 0.003	1.247	1.261	1.394	0.798
		50	0.697 ± 0.011	0.669	0.725	1.155 ± 0.028	1.085	1.225	1.435	0.866
	Purple	20	2.894 ± 0.063	2.738	3.050	1.446 ± 0.039	1.348	1.543	0.346	0.692
		35	1.960 ± 0.022	1.905	2.014	1.441 ± 0.009	1.419	1.463	0.510	0.694
		50	1.974 ± 0.021	1.922	2.026	1.252 ± 0.007	1.234	1.271	0.507	0.798
	White	20	1.881 ± 0.026	1.818	1.945	1.055 ± 0.017	1.012	1.097	0.532	0.948
		35	1.568 ± 0.024	1.508	1.629	1.011 ± 0.027	0.944	1.078	0.638	0.989
		50	0.897 ± 0.005	0.885	0.908	1.105 ± 0.004	1.095	1.116	1.115	0.905
*SG*	Orange	20	5.205 ± 0.052	5.077	5.333	5.837 ± 0.032	5.757	5.916	0.192	0.171
		35	5.485 ± 0.081	5.284	5.687	5.186 ± 0.088	4.968	5.404	0.182	0.193
		50	4.833 ± 0.027	4.766	4.901	4.683 ± 0.083	4.477	4.890	0.207	0.214
	Pink	20	4.866 ± 0.080	4.668	5.064	6.683 ± 0.116	6.396	6.970	0.205	0.150
		35	5.030 ± 0.070	4.855	5.205	5.079 ± 0.025	5.017	5.141	0.199	0.197
		50	5.189 ± 0.084	4.982	5.397	4.038 ± 0.085	3.826	4.249	0.193	0.248
	Purple	20	27.766 ± 0.633	26.194	29.337	4.553 ± 0.019	4.506	4.600	0.036	0.220
		35	24.283 ± 0.541	22.940	25.626	3.073 ± 0.019	3.027	3.119	0.041	0.325
		50	12.961 ± 0.194	12.480	13.441	4.782 ± 0.107	4.516	5.048	0.077	0.209
	White	20	9.361 ± 0.147	8.995	9.727	7.469 ± 0.073	7.287	7.651	0.107	0.134
		35	6.670 ± 0.079	6.473	6.867	7.276 ± 0.070	7.103	7.449	0.150	0.137
		50	6.589 ± 0.105	6.327	6.850	6.866 ± 0.067	6.700	7.033	0.152	0.146

C.I.—confidence interval.

**Table 2:** Verification of the Peleg models.

		Temperature	χ^2^	RMSE	MBE	MPE	SSE	AARD	r^2^
WL	Orange	20	3.81 × 10^−6^	1.69 × 10^−3^	−6.69 × 10^−5^	0.261	1.14 × 10^−5^	0.261	1.000
		35	8.91 × 10^−5^	8.18 × 10^−3^	9.58 × 10^−5^	1.032	2.67 × 10^−4^	1.032	0.999
		50	1.71 × 10^−4^	1.13 × 10^−2^	9.14 × 10^−5^	1.300	5.13 × 10^−4^	1.300	0.999
	Pink	20	6.12 × 10^−6^	2.14 × 10^−3^	6.26 × 10^−5^	0.335	1.84 × 10^−5^	0.335	1.000
		35	2.67 × 10^−4^	1.41 × 10^−2^	1.14 × 10^−4^	1.728	8.00 × 10^−4^	1.728	0.998
		50	9.37 × 10^−6^	2.65 × 10^−3^	−2.33 × 10^−5^	0.300	2.81 × 10^−5^	0.300	1.000
	Purple	20	3.22 × 10^−5^	4.91 × 10^−3^	−2.46 × 10^−4^	1.144	9.66 × 10^−5^	1.144	0.999
		35	4.33 × 10^−4^	1.80 × 10^−2^	−5.55 × 10^−4^	3.381	1.30 × 10^−3^	3.381	0.993
		50	4.29 × 10^−4^	1.79 × 10^−2^	−6.71 × 10^−4^	3.156	1.29 × 10^−3^	3.156	0.994
	White	20	3.19 × 10^−5^	4.89 × 10^−3^	−2.14 × 10^−4^	0.775	9.57 × 10^−5^	0.775	1.000
		35	5.29 × 10^−5^	6.30 × 10^−3^	2.31 × 10^−4^	0.888	1.59 × 10^−4^	0.888	1.000
		50	7.09 × 10^−4^	2.31 × 10^−2^	−3.30 × 10^−4^	2.694	2.13 × 10^−3^	2.694	0.994
SG	Orange	20	2.42 × 10^−7^	4.26 × 10^−4^	−7.08 × 10^−6^	0.273	7.25 × 10^−7^	0.273	1.000
		35	8.13 × 10^−7^	7.81 × 10^−4^	1.69 × 10^−5^	0.474	2.44 × 10^−6^	0.474	1.000
		50	1.98 × 10^−5^	3.86 × 10^−3^	−8.01 × 10^−5^	2.084	5.95 × 10^−5^	2.084	0.997
	Pink	20	3.21 × 10^−5^	4.91 × 10^−3^	5.27 × 10^−5^	3.424	9.63 × 10^−5^	3.424	0.991
		35	2.30 × 10^−6^	1.31 × 10^−3^	−2.56 × 10^−5^	0.760	6.91 × 10^−6^	0.760	1.000
		50	7.70 × 10^−5^	7.60 × 10^−3^	2.16 × 10^−4^	3.939	2.31 × 10^−4^	3.939	0.990
	Purple	20	2.73 × 10^−6^	1.43 × 10^−3^	1.80 × 10^−4^	2.335	8.19 × 10^−6^	2.335	0.999
		35	1.54 × 10^−5^	3.40 × 10^−3^	4.84 × 10^−4^	4.533	4.62 × 10^−5^	4.533	0.995
		50	1.31 × 10^−6^	9.93 × 10^−4^	6.86 × 10^−5^	0.924	3.94 × 10^−6^	0.924	1.000
	White	20	1.01 × 10^−7^	2.76 × 10^−4^	−7.61 × 10^−6^	0.259	3.04 × 10^−7^	0.259	1.000
		35	6.38 × 10^−6^	2.19 × 10^−3^	3.79 × 10^−5^	1.775	1.91 × 10^−5^	1.775	0.998
		50	9.22 × 10^−6^	2.63 × 10^−3^	4.89 × 10^−5^	2.050	2.77 × 10^−5^	2.050	0.997

**Table 3:** Regression coefficients in the GAB model.

	Sweet Potato	Temperature	*Ck*	C.I. (−95%)	C.I. (−95%)	*k*	C.I. (−95%)	C.I. (−95%)	*Xm*	C.I. (−95%)	C.I. (−95%)
*WL*	Orange	20	2.185 ± 0.037	2.094	2.276	0.937 ± 0.008	0.918	0.956	0.012 ± 0.000	0.012	0.012
		35	2.021 ± 0.015	1.983	2.059	0.863 ± 0.009	0.841	0.885	0.043 ± 0.000	0.042	0.044
		50	2.113 ± 0.017	2.070	2.156	0.903 ± 0.021	0.850	0.956	0.017 ± 0.000	0.016	0.018
	Pink	20	1.997 ± 0.047	1.880	2.114	0.855 ± 0.003	0.846	0.864	0.027 ± 0.001	0.026	0.028
		35	2.178 ± 0.039	2.081	2.275	0.933 ± 0.009	0.912	0.954	0.028 ± 0.000	0.027	0.029
		50	2.205 ± 0.024	2.145	2.265	0.944 ± 0.012	0.914	0.974	0.004 ± 0.000	0.004	0.004
	Purple	20	2.223 ± 0.026	2.158	2.288	0.955 ± 0.018	0.910	1.000	0.021 ± 0.000	0.020	0.022
		35	2.168 ± 0.047	2.050	2.286	0.929 ± 0.011	0.901	0.957	0.020 ± 0.000	0.019	0.021
		50	2.257 ± 0.064	2.098	2.416	0.969 ± 0.017	0.927	1.011	0.023 ± 0.000	0.022	0.024
	White	20	2.185 ± 0.014	2.150	2.220	0.938 ± 0.016	0.899	0.977	0.038 ± 0.001	0.035	0.041
		35	2.162 ± 0.033	2.080	2.244	0.927 ± 0.011	0.900	0.954	0.037 ± 0.001	0.035	0.039
		50	2.297 ± 0.060	2.148	2.446	0.980 ± 0.025	0.917	1.043	0.017 ± 0.000	0.016	0.018

C.I.—confidence interval.

**Table 4:** Verification of the GAB models.

Sweet Potato	Temperature	χ^2^	RMSE	MBE	MPE	SSE	AARD	r^2^
Orange	20	4.26 × 10^−3^	5.65 × 10^−2^	7.99 × 10^−3^	0.688	1.28 × 10^−2^	0.688	1.000
	35	2.69 × 10^−2^	1.42 × 10^−1^	4.67 × 10^−2^	2.090	8.08 × 10^−2^	2.090	0.999
	50	1.11 × 10^−2^	9.11 × 10^−2^	3.66 × 10^−2^	2.201	3.32 × 10^−2^	2.201	1.000
Pink	20	1.44 × 10^−2^	1.04 × 10^−1^	1.67 × 10^−2^	0.848	4.32 × 10^−2^	0.848	1.000
	35	9.31 × 10^−3^	8.36 × 10^−2^	2.11 × 10^−2^	1.131	2.79 × 10^−2^	1.131	1.000
	50	1.83 × 10^−3^	3.70 × 10^−2^	1.21 × 10^−2^	0.709	5.48 × 10^−3^	0.709	0.999
Purple	20	9.90 × 10^−4^	2.73 × 10^−2^	−2.41 × 10^−3^	0.245	2.97 × 10^−3^	0.245	0.999
	35	4.78 × 10^−4^	1.89 × 10^−2^	3.33 × 10^−3^	0.191	1.44 × 10^−3^	0.191	1.000
	50	1.78 × 10^−3^	3.65 × 10^−2^	5.45 × 10^−3^	0.488	5.33 × 10^−3^	0.488	0.999
White	20	7.52 × 10^−3^	7.51 × 10^−2^	2.16 × 10^−2^	1.059	2.26 × 10^−2^	1.059	1.000
	35	8.13 × 10^−3^	7.81 × 10^−2^	1.82 × 10^−2^	1.166	2.44 × 10^−2^	1.166	1.000
	50	3.71 × 10^−4^	1.67 × 10^−2^	−1.34 × 10^−3^	0.185	1.11 × 10^−3^	0.185	1.000

**Table 5:** Activation energy for sweet potato samples for the first and second drying periods (kJ/mol).

Sweet Potato Sample	Orange	Pink	Purple	White
*E_a_*	12.873	15.915	16.652	7.096
*SD*	0.149	0.262	0.207	0.065
C.I (−95%)	12.502	15.265	16.137	6.934
C.I (+95%)	13.244	16.565	17.167	7.258

## Data Availability

The original contributions presented in the study are included in the article, further inquiries can be directed to the corresponding author.
